# Fluxion Dilutions

**Published:** 1882-04

**Authors:** Eldridge C. Price

**Affiliations:** Baltimore, Md.


### FLUXION DILUTIONS.

ELDRIDGE C. PRICE, M. D., BALTIMORE, MD.

Had I introduced the subject of fluxion dilutions into this journal, I should certainly feel a delicacy about continuing the argument further, but since I was herein attacked by Dr. Guernsey, I feel that I have a right to ask a final hearing.

The apparent delay in concluding the subject has been caused by various obstacles of too great importance not to be surmounted before writing this finale.

That the so-called school of Homœopathy is shattered by the schisms of theorists, is a fact to be deplored, but nevertheless it *is* a fact. As my views upon this subject have been expressed elsewhere, I will not afflict my readers with a repetition of them in this connection.

That my friend Dr. Guernsey saw fit to criticise so severely my opinions and statements of fact, published in the *Hahnemannian* for March, 1881, I sincerely regret, for facts remain unchanged even amidst the most wordy warfare.

I have simply acted upon the defensive, and am still so acting; if, therefore, my *parries* should bear the semblance of home-*thrusts*, for the sake of my friend in arms, I hope he may not be unprepared to keep his guard.

As an initiatory feint, I will thank my friend for the historical information given me concerning Dr. Swan's mode of preparing his dilutions from high *potencies.* Not being a subscriber to the defunct *Organon*—which Dr. G. gives as his authority—I must ask forgiveness for the grave (?) ignorance of which I have been guilty. Nevertheless, I will make bold to suggest that my original argument was based upon the preparations and comparisons of Dr. S. A. Jones, and not upon those of Swan, Fincke, or Skinner. From his premises, therefore, Dr. Jones is correct; *ergo*, so must be the writer.

Dr. Guernsey remarks that it is "a strange coincidence, that of all my high dilutions, a little drug substance should have remained in each." Truly—if such be the case—it is; I can but assent.

My friend says: "Hahnemann's principle was to give to each patient *just enough* medicine to effect a cure."

I presume there is no man, of whatever pathy, who will deny this truism; but when we attempt to dictate the precise *quantum sufficit*, when we attempt to circumscribe the logic of hundreds of individuals by our own, then we had better lower the point of our foil and call a truce, for where one foe stood there we will find a legion. The repetition of dose is another vexed question, even among our altissimo-dilutionists. How strange it is that two minds of analogous construction, or that possess a similar appreciation of certain combinations of circumstances, should differ so radically upon some allied subject. Dr. Guernsey repeats his doses but seldom, and strongly disapproves of frequent repetition, while Dr. Swan will repeat a CMM as often as every half-hour. Psychology is yet in its infancy.

There are some physicians who are willing to let each man use the preparation he prefers. These men call white, white, and only protest when shades of color are substituted for this fundamental combination.

Here is something that may be of interest to Dr. Guernsey, which I have taken pains to transcribe from the *N. Y. Med. Times* for January, 1882 (a most excellent journal, by the way), viz.:

"Dr. Arndt, in a recent issue of *The Medical Counsellor*, makes a careful analysis of a large number of well-authenticated clinical reports, representing the various epochs in the history of Homœopathy. After going minutely over the cases claimed to have been cured by the crude drug and the various alterations, the following conclusions are reached:

"1. The *great* bulk of homœopathic practice, from the time of Hahnemann to the present day, has been done with low attenuations.

"2. The prescriptions of low-dilutionists show *at least* as much skill in prescribing correctly and in individualizing closely as do the prescriptions of high-dilutionists.

"3. It is a fallacy to presume that the present position of Homœopathy is due to the labors of men who advocate the exclusive use of high potencies in the treatment of the sick. History proves the contrary.

"4. High-dilutionism, as such, is not gaining in the number of its converts in the ratio with which the membership of the homœopathic school is increasing. An examination of the cases reported as cured by high-dilutions show that a comparatively small number of men furnish an unusually large number of clinical verifications and reports, and that high-dilutionism is kept prominent before the profession by the organized and persistent labors of a small but determined minority.

"5. It is good practice, established by the example of our best men, to repeat the dose at regular and appropriate intervals.

"6. Crude drugs may, and do, act homœopathically, curing promptly cases to which they stand in homœopathic rapport. The use of crude *Quinine* or of any other drug in a crude form, in a case the symptoms of which bear the appropriate degree of similarity to the pathogenetic symptoms of the drug, whether the case be one of intermittent fever or belong to any other disease-group, may *possibly* not be followed by as prompt curative action as if the drug were given in a diluted or potentized form, but the practice is undoubtedly legitimate, and it may be unavoidable and imperative. Under no circumstances does the administration of quinine, or any other drug, in a crude form, constitute a violation of strict homœopathic principles, so long as the conditions above stated are met. The practice of our strongest men, living or dead, and the accummulated clinical evidence of more than fifty years of practice prove the correctness of this position."

Relative to the essentials in the process of potentization, I will say nothing further than to allude to Dr. Guernsey's reference to Hahnemann's "Chronic Diseases." His quotations do not in the slightest militate against what I have written. I did not deny the necessity of dilution any more than I did the necessity of friction. I will repeat a statement made in my last article in this journal: "To make a *potency*, friction is a necessary factor; to make a *dilution* it is not. The preparations of Fincke and Swan are therefore dilutions, because they lack the friction element." *

In conclusion I will propound a question for Dr. Guernsey, Swan, Fincke, or any one else who can answer satisfactorily:

What means are used to prevent the minerals found in Croton or other undistilled waters—used as a menstrum for dilution—from modifying the action of fluxion dilutions?

---

### Open Letter to Prof. Gustav Jaeger, from Prof. A. Butlerow, Member of the Imperial Academy of Sciences, at St. Petersburg, etc., etc.

[In The Homœopathic Physician, Vol. I, Nos. 1 and 7, we made the following remarks:

"Judging from the proofs which science is rapidly accumulating for us, we may say positively, that in the course of time, allopathy

* While we are always glad to publish papers discussing posology, we cannot see that Dr. Price advances any arguments against high potencies. *Assertions prove nothing.*—Editor H. P.

